# Multi-layer internal limiting membrane plug technique for management of large full-thickness macular holes

**DOI:** 10.1186/s40942-022-00428-7

**Published:** 2022-11-14

**Authors:** Ehab N El Rayes, Mahmoud Leila, Panagiotis Stavrakas

**Affiliations:** 1 Retina department, Research Institute of Ophthalmology (RIO), 2, Al-Ahram Street, Giza, Egypt; 2 University of Patras Medical School, Patras, Achaea, Greece

**Keywords:** Large macular holes, Secondary macular holes, Techniques for ILM peeling, ILM plug

## Abstract

**Background:**

To evaluate the efficacy of the multi-layer internal limiting membrane plug (MIP) technique in promoting the closure of large full-thickness macular holes (FTMH) and improvement of visual function.

**Methods:**

A prospective interventional non-comparative consecutive case series including patients with surgically naïve large FTMH whether primary or secondary. All macular holes were > 400 µm. All patients had 23-gauge pars plana vitrectomy (PPV), MIP technique, and sulfur hexafluoride (SF6) 20% gas tamponade. The main outcome measures were the closure of the hole, improvement of best-corrected visual acuity (BCVA), and detection of complications that might develop due to surgery.

**Results:**

The study included 15 eyes of 15 patients. The mean age was 44 years (range 10–68; SD 21.5). Primary FTMH constituted 53% of cases. The mean pre-operative minimum linear diameter (MLD) was 702 µm (range 401–1068 µm; SD 154). The mean duration of the macular hole was 6 months (range 1–24; SD 6). The mean pre-operative BCVA was 0.06 decimal units (range 0.01–0.1; SD 0.03). Post-operatively, the macular hole was closed in all patients. U- and V- type closure developed in 93% and 7% of patients, respectively. None of the patients developed W-type closure. Post-operatively, the mean post-operative BCVA was 0.2 decimal units (range 0.05–0.5; SD 0.1). The mean improvement was 5 lines of vision. The mean postoperative follow-up period was 4 months (range 1–10; SD 2.5). None of the patients developed complications attributed to the surgical technique described.

**Conclusion:**

MIP technique is effective in promoting macular hole closure and improvement of visual function in large FTMH.

**Supplementary Information:**

The online version contains supplementary material available at 10.1186/s40942-022-00428-7.

## Background

Large full-thickness macular holes (FTMH) are prone to become refractory to pars plana vitrectomy (PPV), and conventional internal limiting membrane (ILM) peel described by Kelly and Wendel in 1991, and by Eckardt in 1997, respectively [1–6]. The term large hole has been reserved classically to FTMH > 400 µm in diameter per biomicroscopic classification by Gass [7, 8], and per the classification of the international vitreomacular traction study group [9]. Recently, the cut-off diameter of large FTMH associated with lower hole closure rates after conventional surgery has been raised to ≥ 500 µm [10, 11]. Large FTMH could be primary, persistent, or recurrent after surgery, or secondary to an underlying pathology such as trauma, high myopia, solar injury, traction due to proliferative diabetic retinopathy (PDR), or de-roofing of cystoid macular edema (CME) caused by retinal vascular diseases [12]. Macular holes in the latter category share in common the existence of pathogenetic factors that are significant riders to large size and to the classic pathogenesis of primary FTMH based on transvitreal anteroposterior and tangential traction forces as described by Gass [7, 8, 13]. Factors like tissue loss in traumatic FTMH [14–17], outward traction exerted on the macular area by posterior staphyloma in myopic traction maculopathy (MTM) [18–21], and atrophic and ischemic changes in the macular area secondary to retinal vascular diseases [22, 23] perpetuate gaping of the hole and render it either resistant to closure or liable to open-flat closure despite relief of anteroposterior and tangential traction by PPV and conventional ILM peel. In the present series we propose a novel surgical approach, in which we gave the term multi-layer internal limiting membrane plug (MIP) technique for management of large FTMH. The technique consists of peeling a whole area of ILM that is at least double the hole size without avulsing it from the edges of the hole. The ILM is then stacked in multiple layers forming an ILM plug to close the hole and is resilient to displacement. The rationale of the technique is that residual attachment of the ILM to the hole edges and the remnants of the footplates of Müller cells on the retinal side of the ILM would incite glial cell proliferation to close the hole [24]. The aim of the present work is to evaluate the efficacy of this technique in promoting the closure of FTMH, and the improvement of visual function.

## Methods

This is a prospective interventional non-comparative consecutive case series conducted in 2 retina tertiary centers in Egypt and Greece during 2021–2022. Inclusion criteria were surgically naïve FTMH > 400 µm in diameter whether primary or secondary. Exclusion criteria were FTMH < 400 µm in diameter, previous conventional ILM peel, media opacity hindering pre-operative evaluation of the macular hole, co-existing retinal detachment due to peripheral hole, or co-existing retinal pathology that would hinder the improvement of vision as age-related macular degeneration, choroidal rupture bisecting the fovea or extensive atrophy of the retinal pigment epithelium (RPE). The main outcome measures were the closure of the hole, improvement of best-corrected visual acuity (BCVA), and detection of complications that might develop due to surgery. All recruited patients received full ophthalmological examination including history taking, measurement of BCVA using decimal notation, assessment of the intra-ocular pressure (IOP) using applanation tonometry, slit-lamp anterior segment examination, fundus biomicroscopy using the Volk area centralis contact lens for evaluation of the macular hole, retinal examination using indirect ophthalmoscopy including scleral indentation, and optical coherence tomography (OCT) imaging. On biomicroscopy, FTMH was defined as a rounded full-thickness defect in the fovea with a surrounding sub-retinal cuff of fluid and yellowish pigments in the base of the hole. On OCT examination, FTMH was defined as a full-thickness defect of the neurosensory retina in the fovea from the ILM to the RPE with elevated edematous edges and sub-retinal fluid. Mean linear diameter (MLD) refers to the shortest horizontal distance between the hole edges. OCT examination was performed using the spectral domain enhanced-depth imaging (EDI–OCT) Spectralis tracking laser tomography, Heidelberg Engineering GmbH, Heidelberg, Germany.

## Surgical technique

All recruited patients had 23-gauge PPV and MIP technique. Three vitreoretinal surgeons from 2 centers (EE, ML, and PS) performed case selection, surgical intervention, and macular hole imaging. The surgical technique consisted of core vitrectomy followed by triamcinolone acetonide-assisted induction of the posterior hyaloid. The surgeon then proceeded to stain the ILM using brilliant blue G 0.025% stain; Dutch Ophthalmic Research Center (DORC), The Netherlands. After aspirating the supernatant stain, the MIP technique was initiated at a point that was distal to the hole by at least double its size using the Grieshaber FINESSE Flex Loop, Alcon, Geneva, Switzerland, or the Tano diamond dusted scraper. First, multiple breaks were created in the ILM in a rosette fashion at 360° degrees around the hole. Afterward, the flap was reflected centripetally towards the hole under perfluorocarbon liquid (PFCL) without avulsing it off the edges and stacked gently over one another using the Flex Loop, Tano scraper, or vitreoretinal forceps to form a multi-layer plug. Additional file 1: Video S1, supplemental digital content 1. The surgeon did not perform any attempt to tuck the flap inside the hole. Finally, air/PFCL exchange was performed followed by air/sulfur hexafluoride (SF6) 20% exchange. All patients were asked to initially adopt a face-down position for at least 2 days and then a reading position for 3 more days.

The post-operative follow-up schedule included visits at 1 day, 1 week, and 1 month then 3-monthly visits whenever applicable. The examination included BCVA and OCT imaging to determine the pattern of hole closure whether U, V, or W. U-pattern closure was defined as complete re-approximation of the hole edges with no intervening bare RPE area. V-pattern closure was defined as re-approximation of the hole edges but with a steep foveal contour reminiscent of a notch and no intervening bare RPE area. W-pattern closure was defined as flattened hole edges against the RPE with no sub-retinal fluid, and the presence of an intervening bare RPE area. All patients who completed ≥ 3 months follow-up- had a microperimetry examination to detect macular sensitivity and fixation [Micropeimeter (MP-3)], Nidek, Japan.

## Results

The study included 15 eyes of 15 patients. Eight patients (53%) were males. The mean age was 44 years (range 10–68; SD 21.5). Eight patients (53%) had primary FTMH, 4 patients (27%) had traumatic FTMH, 2 patients (13%) had MTM and one patient (7%) had FTMH secondary to de-roofing of CME. The mean pre-operative MLD was 702 µm (range 401–1068 µm; SD 154). The mean duration of the macular hole prior to surgical intervention was 6 months (range 1–24; SD 6). Mean pre-operative BCVA was 0.06 decimal units (range 0.01–0.1; SD 0.03). Post-operatively, the macular hole was closed in all patients. U-type closure was detected in 14 patients (93%) and V-type closure was detected in 1 patient (7%). None of the patients developed W-type closure. Post-operatively, all operated eyes had improved BCVA. The mean post-operative BCVA was 0.2 decimal units (range 0.05–0.5; SD 0.1). The mean improvement was 5 lines of vision. The mean post-operative follow-up period was 4 months (range 1–10; SD 2.5). None of the patients developed complications attributed to the surgical technique described. Tables 1 and 2.

**Table 1:** Baseline patients’ characteristics

Baseline characteristics	N (%)
Male	8 (53)
Female	7 (47)
Age, years	
< 20	3 (20)
20–40	3 (20)
40–60	3 (20)
> 60	6 (40)
Pathology	
Primary	8 (53)
Traumatic	4 (27)
Myopic	2 (13)
CME	1 (7)
MLD (µ)	
> 400–600	3 (20)
> 600–800	9 (60)
> 800	3 (20)
Pre-operative BCVA (decimal)	
< 0.05	3 (20)
0.05–0.1	12 (80)
Duration prior to intervention (months)	
< 3	5 (33)
3–6	7 (47)
> 6	3 (20)

*BCVA* best-corrected visual acuity; *CME* cystoid macular edema; *µ* micron; *MLD* minimum linear diameter

**Table 2:** Post-operative anatomical and functional outcome

Post-operative anatomical and functional outcome	N (%)
Post-operative BCVA (decimal)	
0.05–0.1	8 (53)
> 0.1–0.4	6 (40)
> 0.4	1 (7)
Gain in BCVA (lines of vision)	
1–2	1 (7)
> 2–5	10 (67)
> 5	4 (27)
Closure type	
U	14 (93)
V	1 (7)
W	0 (0)
Duration of follow-up (months)	
1–3	9 (60)
> 3–6	3 (20)
> 6	3 (20)

*BCVA* best-corrected visual acuity

### Case presentation

#### Case no. 1

**Fig. 1:**
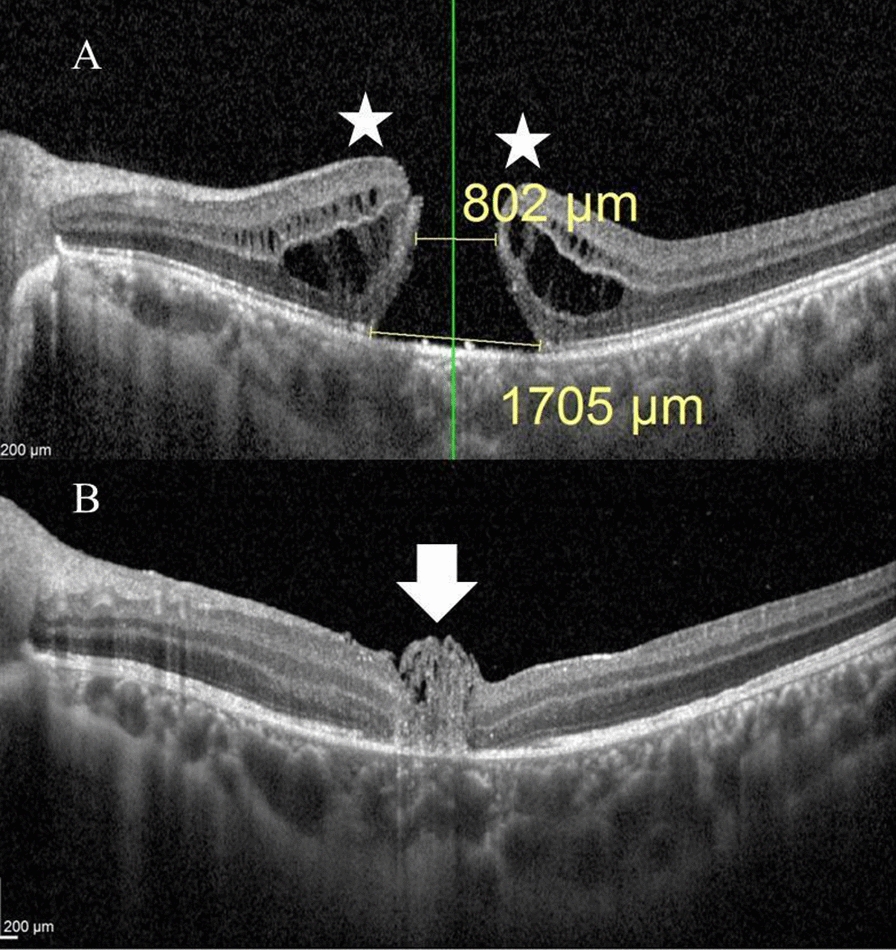
**A** Pre-operative OCT line scan through the macular area in the left eye of a 10-year-old boy with traumatic FTMH. MLD was 802 µm and the base diameter was 1705 µm. Note the pregnant draw-bridge appearance at the hole edges (asterisks). BCVA was 0.05. **B** Post-operative OCT line scan through the macular area in the same eye after 1 month of follow-up. Note the multi-layered ILM plug (arrow) and U-type closure of the hole. BCVA was 0.2

A 10-year-old boy presented with a history of blunt trauma to the left eye by a metal bolt for 2 months. His BCVA was 0.05 decimal units. His anterior segment examination was unremarkable. Fundus examination revealed FTMH. MLD by OCT was 802 µm. Post-operatively, OCT examination revealed U-type closure of the hole. His BCVA at 1 month of follow-up was 0.2 decimal units Fig. 1.

#### Case no. 2

**Fig. 2:**
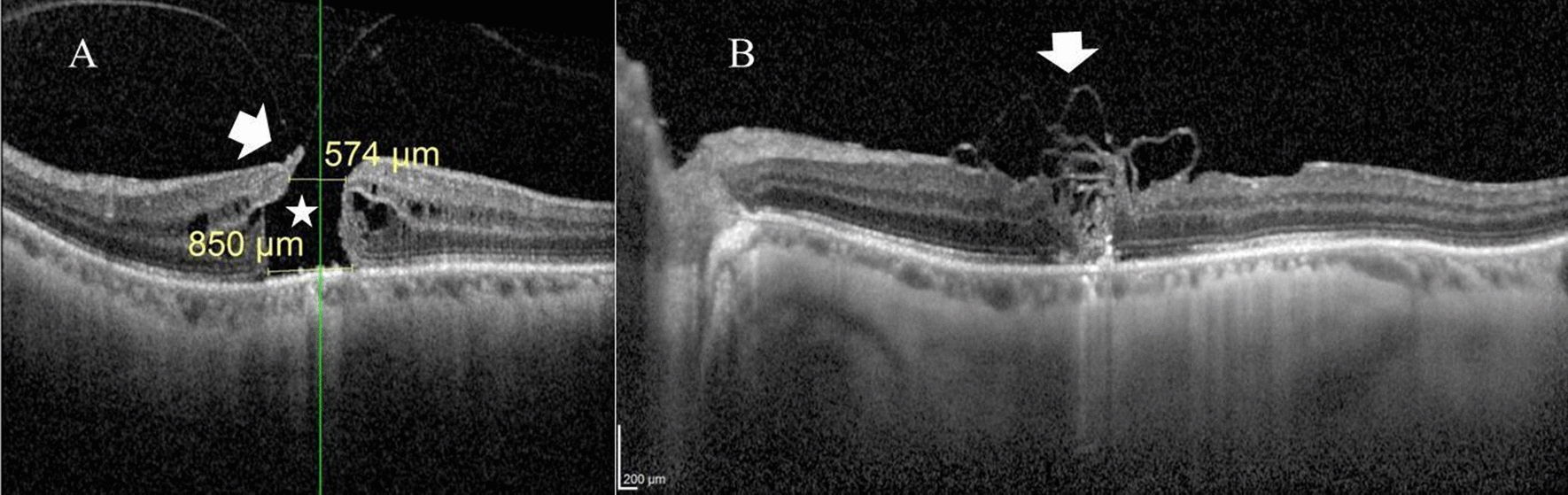
**A** Pre-operative OCT radial scan of the left macula of a 59-year-old female with primary FTMH. MLD was 574 µm and the base diameter was 850 µm. Note the intra-retinal edema at the hole edges (asterisk) and the partially avulsed operculum with focal vitreomacular adhesion (arrow). BCVA was 0.05. **B** Post-operative OCT line scan through the macula after 2 months of follow-up. Note the multi-layered ILM plug (arrow) and U-type closure of the hole. BCVA was 0.2

A 59-year-old female presented with defective vision in the left eye for 3 months duration. Her BCVA was 0.05 decimal units. Her anterior segment examination was unremarkable. Fundus examination revealed FTMH. MLD by OCT was 574 µm. Post-operatively, OCT examination revealed U-type closure of the hole. Her BCVA at 2 months of follow-up was 0.2 decimal units Fig. 2.

#### Case no. 3

**Fig. 3:**
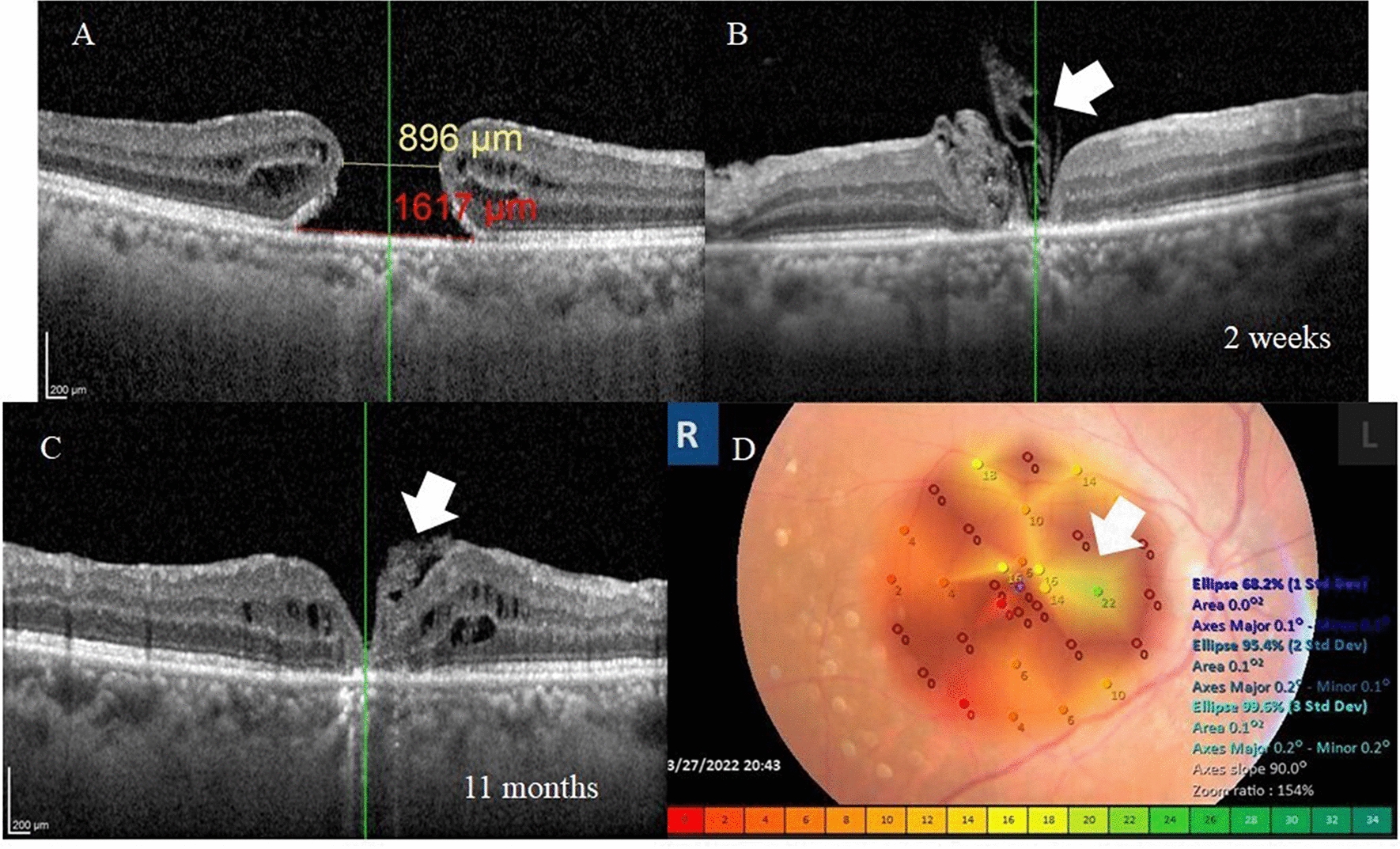
**A** Pre-operative OCT radial scan of the right macula of a 62-year-old female with primary FTMH. MLD was 896 µm and the base diameter was 1617 µm. BCVA was 0.1. **B**, **C** Post-operative OCT at 2 weeks and 4 months, respectively. Note the multi-layered ILM plug (arrow) and the approximation of hole edges with gradual conversion of the hole from W-type at 2 weeks to U-type at 4 months. The final BCVA was 0.2. **D** Microperimetry of the right macular area revealed stable fixation (Fujii classification). BCEA (Bivariate contour ellipse area) values indicated good sensitivity and fixation in the juxta-foveal area (arrow) with depressed both parameters elsewhere

A 62-year-old female presented with a history of defective vision in the right eye of approximately 1-year duration. Her BCVA was 0.1 decimal units. Anterior segment examination revealed pseudophakia with a posterior chamber intra-ocular lens (PCIOL) implanted in the capsular bag. Fundus examination revealed FTMH. MLD by OCT was 896 µm. At 5 months of follow-up, her OCT examination revealed U-type closure of the hole. BCVA was 0.2 decimal units. Microperimetry examination of the right macula at 5 months of follow-up revealed improvement in fixation stability and in juxta-foveal sensitivity Fig. 3.

#### Case no. 4

**Fig. 4:**
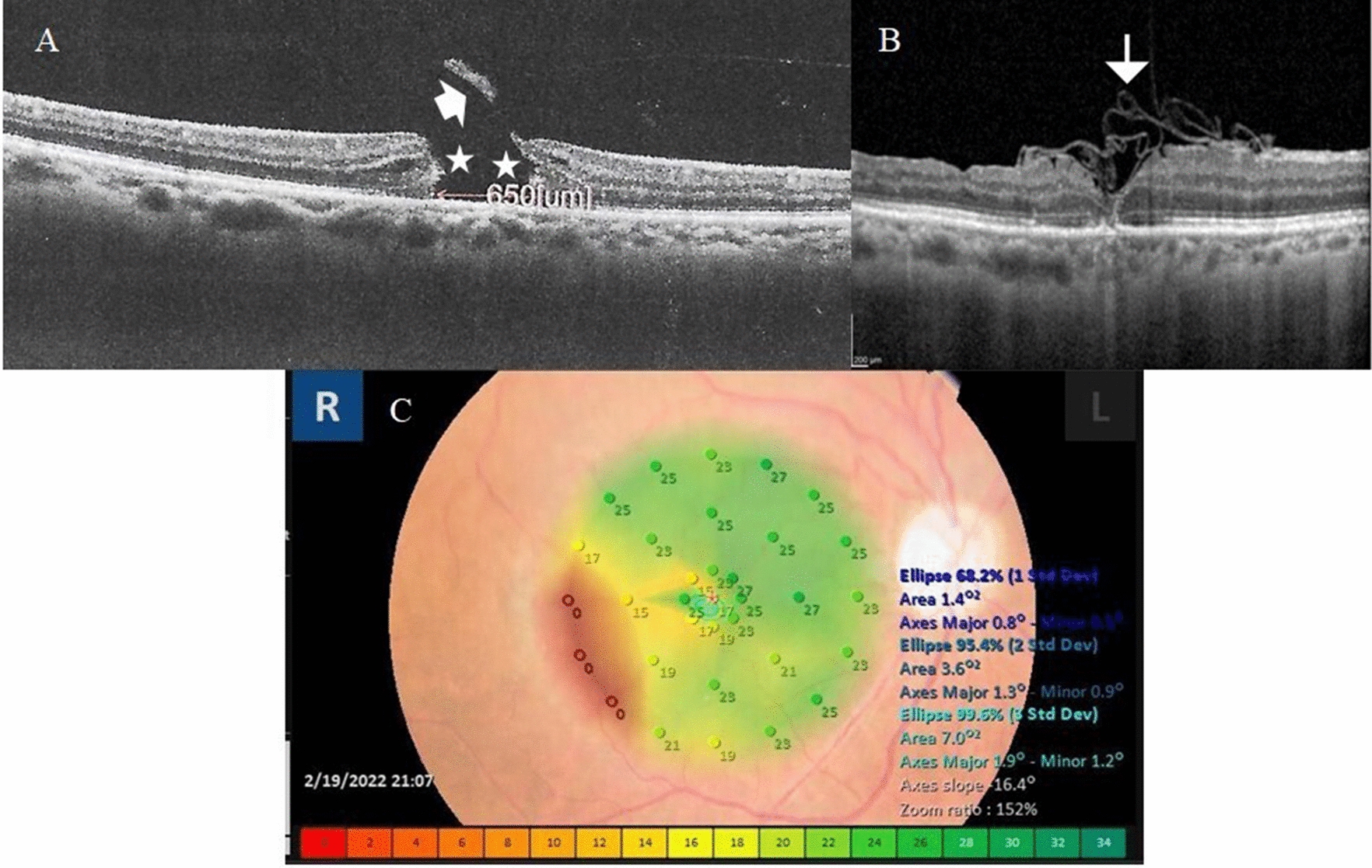
**A** Pre-operative 5-line raster scan of the right macula of a 65-year-old female with primary FTMH. MLD was 650 µm. Note the intra-retinal edema at the hole edges (asterisks). The avulsed operculum was attached to the detached posterior hyaloid (arrow). BCVA was 0.1. **B** Post-operative OCT line scan through the macula after 5 months of follow-up. Note the multi-layered ILM plug (arrow) and U-type closure of the hole. BCVA was 0.4. **C** Microperimetry of the right macular area revealed stable fixation (Fujii classification) and low BCEA values indicating good macular sensitivity and fixation. The dense red color located in the temporal parafoveal area in the color-coded map indicated the lowest macular sensitivity

A 65-year-old female presented with complaints of defective vision in the right eye and the presence of a round black spot obscuring the center of her visual field for one month. Her BCVA was 0.1 decimal units. Her anterior segment examination revealed pseudophakia with PCIOL located inside the capsular bag. Fundus examination revealed FTMH. MLD by OCT was 650 µm. Post-operatively, OCT examination revealed U-type closure of the hole. Her BCVA at 7 months of follow-up was 0.3 decimal units. Microperimetry examination of the right macula at 7 months of follow-up revealed significant improvement in macular sensitivity and fixation stability Fig. 4.

#### Case no. 5

**Fig. 5:**
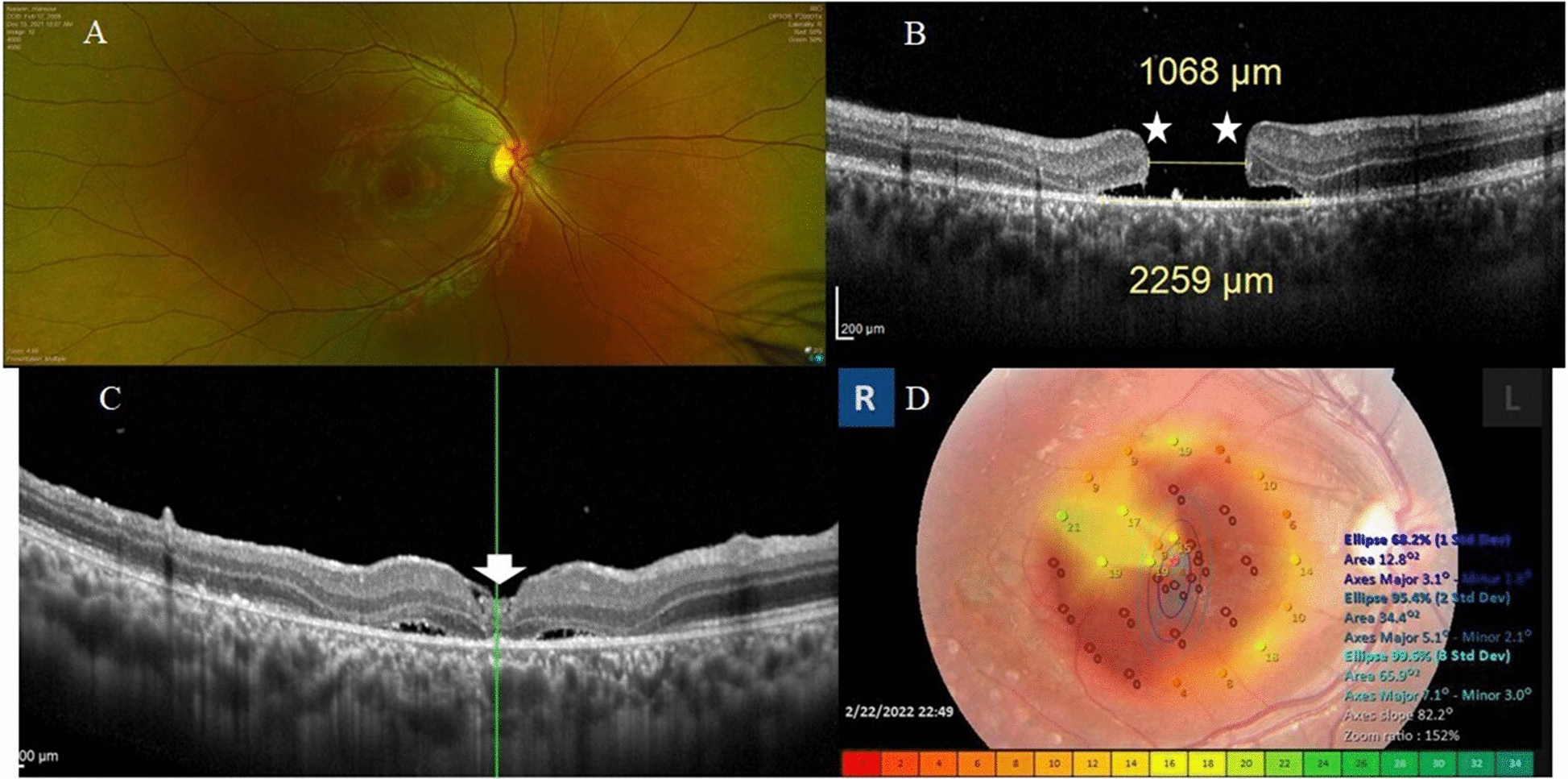
**A** Pre-operative color photo of the fundus of the right eye of a 14-year-old boy with traumatic FTMH. Note the sheer size of the hole, which was approximately ¾ disc diameter, the yellowish pigmentation in the base of the hole, and the sub-retinal cuff of fluid. **B** OCT image of the macula in a radial scan mode. Note the pregnant draw-bridge configuration of the edges of the hole (asterisks). MLD was 1068 µm. The base diameter was 2259 µm. BCVA was 0.05. **C** Post-operative line scan of the macula after 2 months of follow-up. Note the ILM plug (white arrow), and the U-type closure of the hole. BCVA was 0.1. **D** Microperimetry of the right macular area revealed unstable fixation (Fujii classification) and high BCEA values indicating poor macular sensitivity and fixation. Note the dense central scotoma in the color-coded map

A 14-year-old boy presented with complaints of defective vision in the right eye. The parents gave a history of blunt trauma with a closed fist 3 months ago. His BCVA was 0.05 decimal units. Fundus examination revealed FTMH. MLD by OCT was 1068 µm. Post-operatively, OCT examination revealed U-type closure of the hole. His BCVA at 2 months of follow-up was 0.1 decimal units. Microperimetry examination of the right macula at 2 months of follow-up revealed low macular sensitivity and low fixation stability Fig. 5.

## Discussion

PPV and ILM peel remains the standard surgical approach for the management of macular holes. However, this technique did not yield favorable results in terms of closure rates for large macular holes. Although relieving the anteroposterior and tangential traction forces by the fore-mentioned surgical approach is pivotal in promoting hole closure, this cannot fully counteract the multifactorial pathogenesis of large-size primary and secondary macular holes that perpetuate gaping of the hole edges [1–3, 14–23]. Hence, a complementary surgical rider is required to trigger the proliferation of glial cells to close the hole. Michalewska et al. [25] described the inverted ILM flap technique that entailed using the ILM as a scaffold for the proliferating glial cells and to further enhance the healing process by residual foot plates of Müller cells on its retinal surface. In the present study, we deployed the MIP technique. The fundamental difference between our technique and that of Michalewska was that we did not attempt to tuck the ILM flap inside the hole or manipulate the edges of the hole. The rationale was to avoid inflicting damage on the RPE in the base of the hole and to the edges of the hole. Instead, we created multiple layers of ILM and stacked them to cover the hole one layer after another. We found that the resultant multi-layered flap got sucked inside the hole by the negative pressure created once the hole was sealed, eventually acting as a plug. This plug would provide the desired scaffold effect in addition to replenishing the hole with Müller cells residues. We had a macular hole closure rate of 100%, of which 93% was a U–type closure using single surgery. The final mean BCVA improved by 5 lines, nevertheless, the overall visual acuity improvement was marginal as 87% of operated eyes had final BCVA ≤ 0.2. Only 1 patient (7%) achieved a BCVA of 0.5. It is worthy of note that almost 50% of operated eyes in this series had FTMH secondary to a primary pathology that might have compromised the functional outcome. In comparison, Michalewska et al. [25] reported an initial closure rate of 98% using the inverted ILM flap technique and significant improvement of BCVA. The authors recruited exclusively patients with idiopathic macular holes (IMH). The mean MLD was 759 µm. Another series by Michalewska et al. [26] compared the inverted ILM flap technique to the temporal inverted ILM flap technique in 2 groups with FTMH and mean MLD of 533 µm, and 544 µm, respectively. They reported an initial hole closure rate of 93% for either of the 2 techniques. U-type closure was present in 62% and 71% of cases in the inverted ILM flap technique and the temporal inverted ILM flap technique, respectively. The authors reported significant improvement in BCVA using both techniques without a statistically significant difference between the 2 techniques. The study excluded all patients with high myopia and PDR. Casini et al. [27] compared the inverted ILM flap technique to a modified ILM flap technique without extra-manipulation of the flap in which the authors relied on flattening the flap against the hole by the inflowing air during fluid/air exchange. The authors excluded all macular holes secondary to trauma, myopia, or PDR. The mean MLD in both groups was 561 µm, and 603 µm, respectively. They reported initial hole closure rates of 97.6% and 97.5% for the inverted ILM flap technique and the modified ILM flap technique, respectively. U-type closure was present in 77.5% and 66.67% of both groups, respectively. BCVA improved significantly using both techniques but without a statistically significant difference between the 2 techniques. Shin et al. [28] described a single-layered inverted ILM flap technique. The authors had an 83% closure rate after primary surgery. They reported migration of the single-layered ILM in one of their cases. A key success for the MIP technique is the creation of an ILM flap that is at least double the size of the macular hole. The large surface area of the resultant flap compared to the size of the hole facilitates maneuverability and allows stacking the flap in a multi-layer configuration. We found that the layers of ILM tend to stick to each other forming a multi-layered plug that sealed the hole effectively and was resilient to displacement intra-operatively during air/PFC exchange or dislodgement post-operatively as had happened in other techniques used by the fore-mentioned authors [25, 26, 28]. Moreover, the centripetal force exerted while reflecting the flap towards the hole promotes atraumatic approximation of the edges of the hole. Accordingly, we had not had a single case of flap migration post-operatively and we were able to achieve a 100% closure rate. An important point of strength in the present series is that approximately 50% of our patients had FTMH secondary to other pathologies that could hinder hole closure. Nevertheless, we had not had a single case of W-type closure and only 1 case of V-type closure, which emphasizes the efficiency of the MIP technique in promoting hole closure. In addition, in our study, the mean MLD was 702 µm compared to a mean MLD of 400.8 µm, and a median MLD of 558 µm in the BEAVRS macular hole outcome group study [10], and the Manchester large macular hole study [11], respectively. These studies concluded that conventional ILM peel is associated with lower success rates for macular hole diameters exceeding 500 µm, and 630 µm, respectively, which further corroborates the rationale for using the MIP technique for large-size FTMH. The limitations of the present study include its limited sample size and inhomogeneity of the sample in terms of pathogenesis. Therefore, we can only recommend the MIP technique as promising surgical management for large FTMH awaiting prospective studies with a larger sample size comparing MIP to other surgical techniques.

## Conclusion

MIP technique is effective in promoting macular hole closure and improvement of visual function in large FTMH.

## Supplementary Information

**Additional file 1: Video S1.** Surgical technique of MIP.

## Data Availability

All data pertaining to the present study are confidential. Access to these data will be granted exclusively to people or entities who meet the criteria for access to confidential data and only upon written request. All requests should be addressed to the corresponding author: Professor Ehab N. El Rayes. 35 Salah Salem St., (El Borg), Suite 702, El-Obour bldg. Cairo 11,371, Egypt.

## Abbreviations

BCEA: Biavariate contour elipse area
BCVA: Best-corrected visual acuity
CME: Cystoid macular edema
DORC: Dutch Ophthalmic Research Center
EDI-OCT: Enhanced depth imaging–optical coherence tomography
ELM: External limiting membrane
EZ: Ellipsoid zone
FTMH: Full-thickness macular hole
IOP: Intra-ocular pressure
ILM: Internal limiting membrane
IMH: Idiopathic macular hole
IS/OS: Inner segment/outer segment layer
MIP: Multi-layer internal limiting membrane plug
MLD: Minimum linear diameter
µm: Micrometer
MTM: Myopic traction maculopathy
OCT: Optical coherence tomography
PDR: Proliferative diabetic retinopathy
PFCL: Perfluorocarbon liquid
PPV: Pars plana vitrectomy
RIO: Research Institute of Ophthalmology
RPE: Retinal pigment epithelium
SD: Standard deviation
SF6: Sulfur hexafluoride
